# Biogeographical characterization of *Saccharomyces cerevisiae* wine yeast by molecular methods

**DOI:** 10.3389/fmicb.2013.00166

**Published:** 2013-06-24

**Authors:** Rosanna Tofalo, Giorgia Perpetuini, Maria Schirone, Giuseppe Fasoli, Irene Aguzzi, Aldo Corsetti, Giovanna Suzzi

**Affiliations:** Faculty of BioScience and Technology for Food, Agriculture and Environment, University of TeramoMosciano Sant’Angelo, TE, Italy

**Keywords:** *Saccharomyces cerevisiae*, wine, biogeography, molecular methods, *terroir*

## Abstract

Biogeography is the descriptive and explanatory study of spatial patterns and processes involved in the distribution of biodiversity. Without biogeography, it would be difficult to study the diversity of microorganisms because there would be no way to visualize patterns in variation. *Saccharomyces cerevisiae*, “the wine yeast,” is the most important species involved in alcoholic fermentation, and in vineyard ecosystems, it follows the principle of “everything is everywhere.” Agricultural practices such as farming (organic *versus* conventional) and floor management systems have selected different populations within this species that are phylogenetically distinct. In fact, recent ecological and geographic studies highlighted that unique strains are associated with particular grape varieties in specific geographical locations. These studies also highlighted that significant diversity and regional character, or ‘*terroir*,’ have been introduced into the winemaking process via this association. This diversity of wild strains preserves typicity, the high quality, and the unique flavor of wines. Recently, different molecular methods were developed to study population dynamics of *S. cerevisiae* strains in both vineyards and wineries. In this review, we will provide an update on the current molecular methods used to reveal the geographical distribution of* S. cerevisiae *wine yeast.

## INTRODUCTION

One of the most important issues in ecological studies is the determination of microbial biodiversity distribution and thus the understanding of whether microorganisms are cosmopolitan or endemic to a specific area or host (Ramette and Tiedje, 2007). Biogeography is the discipline that studies the distribution of biodiversity over space and time (Martiny et al., 2006). During the 18^th^ century, biologists applied this approach to study the geographic distribution of plant and animal diversity, and only more recently, interest in the geographic distribution of microorganisms has increased. The aim of microbiogeography is to reveal where microorganisms live, their abundance and distribution, and their diversity over different taxonomic and spatial scales. In fact, genetic distance may be correlated with geographic distance and/or environmental characteristics (e.g., salinity, depth, latitude; Schuller et al., 2012). The scope of microbiogeography also encompasses the understanding of the processes generating and maintaining the distribution of microorganisms (Ramette and Tiedje, 2007). Other goals of this field are to propose and evaluate theories regarding the creation and evolution of such diversity patterns in the environment (Ramette and Tiedje, 2007). The first paradigm in microbial biogeography, “*Alles is overal, maar het milieu selecteert*” (“everything is everywhere, but the environment selects”) was offered by Baas Becking more than 70 years ago (O’Malley, 2008). This appealing idea was based on the small size and high dispersal potential of microorganisms and their large populations and low presumed extinction rates (Ramette and Tiedje, 2007). However, even if the field of microbial biogeography is not new, the determinism of microbial diversification and distribution has been poorly documented and is not well understood. This may partly be due to the natural properties of microorganisms (e.g., their small size, which makes access within different environmental matrices difficult, their huge diversity, and the complexity of precisely defining their species) and the lack of an adequate sampling strategy. Recently, the development of new molecular tools has partially resolved these limitations; in fact, recent developments have allowed the survey of uncultivated microorganisms in the environment and the characterization of microbial community structure (Christen, 2008). Furthermore, these tools are now generally automated and allow the moderate throughput essential to studies involving the characterization of numerous samples of different origins. In particular, the use of DNA, RNA and protein sequences for the construction of evolutionary trees has allowed a better understanding of the way in which biodiversity was generated. Hence, the application of molecular phylogenetic methods to study natural microbial ecosystems has resulted in the unexpected discovery of many evolutionary lineages (Suzzi, 2011). Moreover, metagenomic and metatranscriptomic approaches will allow not only the dissolution of the species concept issue but will also separate the relationship between the notion of species and their spatial distribution. Weiher and Keddy (1995) proposed that a trait-based approach should be the basis of a conceptual model for trait-based community assembly. In particular, traits, not taxon names, are the fundamental units of biodiversity and biogeography. Microorganisms that show similar traits share the same ecological niche. Therefore, the principal challenge of microbiology is to identify the main genetic variants inducing phenotypic variation and niche adaptation (Cubillos et al., 2011).

**FIGURE 1:**
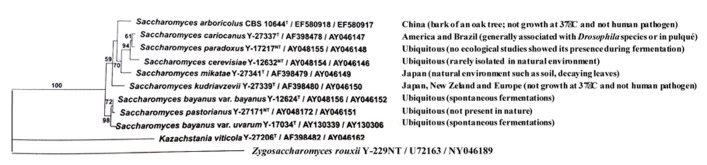
**Geographical characteristics and phylogenetic relationship among *Saccharomyces* species based on the combined sequence analysis of the D1/D2 LSU rRNA gene and ITS (modified from Replansky et al., 2008; Kurtzman et al., 2011)**.

Yeasts of the *Saccharomyces sensu stricto* species complex (**Figure 1**) are able to convert sugar into ethanol and CO_2_ via fermentation. They have been used for thousands of years by mankind for the production of fermented beverages and foods. These yeasts show interesting features that are specific and not found in other genera; for example, they are able to survive in the absence of oxygen by using the fermentation process (Sicard and Legras, 2011). The *Saccharomyces sensu stricto* genus is composed of species showing a level of nucleotide divergence similar to that found between birds and humans (Dujon, 2006). The *sensu stricto* complex is thought to be young; in fact, some studies have suggested that *Saccharomyces* *cerevisiae* diverged from the common ancestor of *Saccharomyces* *paradoxus* and *Saccharomyces* *cariocanus *approximately 5–10 million years ago (Mya), whereas *Saccharomyces* *kudriavzevii. Saccharomyces* *bayanus*, and *Saccharomyces* *mikatae* diverged 10–15, 15–20, and 20 Mya, respectively (reviewed in Replansky et al., 2008).

Recently, Libkind et al. (2011) identified a new species very similar to *Saccharomyces* *bayanus *and called it *Saccharomyces eubayanus* sp. nov., which exists in apparent sympatry in *Nothofagus* (Southern beech) forests in Patagonia. This species is 99.5% identical to the non-*Saccharomyces cerevisiae* portion of the *Saccharomyces* *pastorianus *genome sequence. Because *Saccharomyces pastorianus* and *Saccharomyces bayanus *(a complex hybrid of *Saccharomyces eubayanus*, *Saccharomyces uvarum*, and *Saccharomyces cerevisiae*) are considered to be “a product of the artificial brewing environment with no occurrence in nature,” they may be associated with domestication events and hybrid lineages, whereas *Saccharomyces* *uvarum* and *Saccharomyces* *eubayanus* may be conserved as descriptors of the species.

Recently, the budding yeast *S*. *cerevisiae *has been considered to be an important model for ecological and evolutionary genetics. The ancestor of the *sensu stricto* complex underwent whole-genome duplication. This event was followed by the loss of approximately 90% of the duplicated genes. In fact, comparison of the *S*. *cerevisiae* genome with that of the pre-duplication species *Kluyveromyces waltii* reveals the presence of approximately 500 paralogs among the 5500 genes (Replansky et al., 2008). These duplications may then be subjected to mutations, which may be related to the evolution of new functions or sequence divergence, and inactivation due to the accumulation of non-sense mutations, which leads to relics (Liti and Louis, 2005). The duplicated genes may evolve at different rates, providing new functions. For example, the abilities to grow anaerobically and to produce ethanol and the low- and high-affinity glucose systems may be a consequence of genome duplication and may have offered a competitive advantage against bacteria and other microorganisms.

Scannell et al. (2011) resequenced and reassembled the genomes of *S*. *mikatae*, *S*. *kudriavzevii*, and *S*. *bayanus* and compared them with the *S*. *paradoxus* genome (Liti et al., 2009) and the reference genome of *S*. *cerevisiae* (Goffeau et al., 1996). The authors annotated 5261 sets of genes that are orthologous among all five species and identified 123 genes that could be used as targets of positive selection and may play important roles in ecological specialization. Moreover, these authors underlined that whole-genome duplication still influences yeast evolution and contributes to the genomic and phenotypic differences characterizing *S*. *cerevisiae *and its related species. In addition, the presence of two possible horizontal gene transfers from bacteria was described (Scannell et al., 2011). The possibility of a horizontal genetic exchange from bacteria was also suggested by Wei et al. (2007). The YJM789 genome (a yeast isolated from the lung of an AIDS patient with pneumonia) highlighted a putative horizontal transfer of YJM-GNAT (an unknown gene belonging to the GNAT superfamily related to antibiotic resistance) from bacteria and a potential introgression of a 12-kb sequence of chromosome I from a closely related yeast (Wei et al., 2007).

Recent analyses have shown that yeast hybrids may be more abundant in both natural and industrial environments than previously thought. Indeed, almost 10% of *Saccharomyces* strains previously classified as *sensu stricto *appear to be hybrids of different species (Liti and Louis, 2005). In fact, interspecific hybrid strains, which contain genetic contributions from both *S. cerevisiae *and other *Saccharomyces* spp., may have selective advantages deriving from the combination of desirable traits from both parental species. Recently, several strains involved in winemaking were found to be hybrids between *S*. *cerevisiae* and *S. kudriavzevii* (Gonzales et al., 2006; Erny et al., 2012). Initially, this last species was isolated in Japan, and although Sampaio and Goncalves (2008) also found it in Portugal, it has never been isolated from wine fermentation. However, the Portuguese *S. kudriavzevii *population showed genetic differences compared with the type strain of the species that represents the Japanese population. In wine fermentation, the hybrids exhibit the best properties of both parental species, such as the low-temperature fermentation ability of *S. kudriavzevii *and the high ethanol resistance of *S*. *cerevisiae*. Dunn et al. (2012) analyzed 69 commercial wine yeasts and compared them with other industrial yeasts, wine yeasts, beer yeasts, bread yeasts, and fuel ethanol yeasts. An interspecific hybridization between *S. cerevisiae *and *S. kudriavzevii *in four of the 69 commercial wine strains was observed, and *S. paradoxus *and *S. mikatae *introgression events were detected.

It is unknown when humans began to add selected yeast to make fermented beverages and foods. Such human activities caused hybridization between species and variation of ploidy, which contributed to the evolution of domesticated yeasts.

*Saccharomyces* *cerevisiae* strains are adapted to different niches, so they represent a rich resource for revealing the evolutionary trajectories of a trait because particular molecular profiles may have been selected in specific environments. Moreover, several studies found evidence for a role of geographical isolation in the differentiation of the *S*. *cerevisiae* population in nature, indicating that *S*. *cerevisiae* can be used as model for evolutionary biology and biogeography (Carreto et al., 2008; Replansky et al., 2008; Lidzbarsky et al., 2009; Liti et al., 2009).

Recent resequencing and phylogenetic characterization of multiple *S. cerevisiae* isolates provided evidence of substantial genetic and phenotypic diversity (Liti et al., 2009).

Wang et al. (2012) performed a population genetics analysis of wild Chinese isolates with different ecological and geographical origins. They identified eight new, distinct wild lineages (coded as CHNI-VIII) from a set of 99 Chinese isolates. These lineages were characteristic of specific geographical areas and ecological niches. In particular, these results indicate that a geographically isolated source is important for *S*. *cerevisiae* population differentiation in nature. In fact, this study showed that oak isolates from different regions in northern China clustered into different lineages, and the Chinese oak isolates were clearly separated from those from North America.

Strains of *S. cerevisiae* associated with vineyards and wine production, hereafter referred to as wine strains, often form a genetically differentiated group that is separate from wild strains isolated from soil and oak tree habitats and strains from other fermentation types, such as palm wine, and sake (Fay and Benavides, 2005; Legras et al., 2007; Liti et al., 2009; Schacherer et al., 2009). Several authors have explained these differences as a consequence of domestication. These domestication events were followed by human-associated dissemination of these yeasts throughout the world.

In a recent study, Legras et al. (2007) investigated the possible effects of human history on spreading and selecting this yeast. In particular, they analyzed 651 yeast strains with 56 different origins (beer, bread, palm wine, wine, and rice wine) from five continents. All wine yeasts grouped together and were well separated from the yeast strains of other technological origins. For “non-wine strains,” a relationship between genotype and the isolation source was found. In particular, three Asian groups of strains were identified: the first included the sake yeast group, and the other two contained rice wine and Chinese distillery strains. Regarding the African yeast populations, a Nigerian palm wine group was identified, which also included an Ivory Coast strain. Ghana sorghum beer strains were distinct from palm wine, Burundi cassava and banana strains, suggesting genetic differentiation among African yeast populations (Legras et al., 2007). The wine yeast group contains strains from ancient vine areas (Lebanon, Europe) as well as ‘new world’ recent vineyards, which suggests a migration of wine yeast all over the world. In addition to the historical human transport across the Mediterranean Sea, the phylogenetic analysis obtained clearly supports the hypothesis of a migration pathway along the Danube valley. The way in which wine strains are naturally propagated is still poorly understood: flor yeasts, which grow almost continuously on the surface of wine during the sherry wine process, are likely an example of domestication; in fact, they may present specific features and mutations (Fidalgo et al., 2006). However, for other types of wine strains, we cannot infer such a continuous human control of their culture.

Fay and Benavides (2005) investigated the genetic differences among strains that were of wine origin and those that were not. The population of *S*. *cerevisiae* associated with the wine ecosystem were genetically homogenous. The reduced levels of variation present in winemaking strains may have been the result of a genetic bottleneck, selection for specific traits, or a combination of the two (Hyma et al., 2011).

The population structure of *S*. *cerevisiae* in nature remains obscure. The different *S*. *cerevisiae* isolates are characterized by large genetic and phenotypic variations, providing a powerful tool for quantitative genetic studies (Liti et al., 2009). This apparent variation is likely because some studies were performed on laboratory strains, which are highly adapted to artificial conditions and do not represent the true ecological diversity of the species (Steinmetz et al., 2002; Ehrenreich et al., 2010). Recently, it was demonstrated that environmental factors and the interactions between each organism and its environment influence genomic rearrangements and the evolution of phenotypes (Camarasa et al., 2011; Warringer et al., 2011). In particular, Camarasa et al. (2011) related the metabolic traits of *S. cerevisiae *strains with their origins. These strains were isolated from seven different niches (baker, clinical, fermentation processes, laboratory, vineyard, natural, and commercial wine yeasts). The relationships were established using a statistical approach that allowed the identification of specific features common to all strains belonging to the same niche. Some metabolic differences of strains with different origins are shown in **Table 1**. Phenotypic variation in *Saccharomyces* strains collected from diverse natural habitats, used in industrial processes, and associated with human illness was observed (Kvitek et al., 2008). Phenotypic variation in stress sensitivity and gene expression was also observed. Vineyard isolates survived better in the presence of different stress conditions due to their ability to thrive in more variable natural environments, which facilitated their dispersal into new environments in a manner associated with human interactions (Kvitek et al., 2008). The main approach used to establish a relationships between genotypic variation and phenotypes is mapping of quantitative trait loci (QTL). This technique allowed to map the loci responsible for brewing characteristics in a sake strain, ethanol resistance, xylose utilization for application in the bioethanol industry, acetic acid production, and fermentation performance in wine strains (Borneman et al., 2013).

**Table 1:** Sequenced genomes of wine *Saccharomyces cerevisiae* strains (modified from Borneman et al., 2013).

*S. cerevisiae* strain	Project	Origin	Reference
RM11-1a	Assembly	Vineyard^a^-USA	Wei et al. (2007)
YPS163	Low coverage assembly	Vineyard-Italy	Doniger et al. (2008)
AWRI1631	Assembly	Wine^a^	Borneman et al. (2008)
EC1118	Assembly	Commercial wine yeast^b^	Novo et al. (2009)
AWRI796	Assembly	Commercial wine yeast	Borneman et al. (2011)
Lalvin QA23	Assembly	Commercial wine yeast	
Vin13	Assembly	Commercial wine yeast	
VL3	Assembly	Commercial wine yeast	
YJM269	Assembly	Wine grapes	–
T73	Assembly	Wine-Spain	
Y55	Low coverage assembly	Grape^a^-France	Liti et al. (2009)
L-1528	Low coverage assembly	Wine^a^-Chile	
BC187	Low coverage assembly	Wine^a^-USA	
DBVPG1106	Low coverage assembly	Grapes^a^-Australia	
YIIc17_E5	Low coverage assembly	Wine^a^-France	
Y12	Low coverage assembly	Palm wine^a^-Africa	
DBVPG6044	Low coverage assembly	Bili wine^a^-Africa	
WE372	Raw data only	Wine-South Africa	–
Y12	Raw data only	Palm wine-Africa	

a Haploid derivate of original isolate;

b Haploid sequence representation of diploid strain.

It is well known that the wine yeast, *S. cerevisiae, *plays a major role in the fermentation of grape musts; in fact, it is well adapted to this process (Martini and Vaughan-Martini, 1990; Blondin et al., 2011). In particular, this yeast is adapted to the harsh conditions in grape musts and grapes (high sugar concentration, increasing alcohol concentration, acidity, presence of sulfites, anaerobiosis, and progressive depletion of essential nutrients, such as nitrogen, vitamins, and lipids), and its genome has been modeled, so the understanding of the adaptation phenomenon to the wine environment is a key element in wine yeast genome research (Blondin et al., 2011).

## *Saccharomyces cerevisiae* WINE YEAST

*Saccharomyces cerevisiae* is one of the best model systems used for understanding microbial ecology and evolutionary genetics. Many functional analysis projects have been dedicated to the investigation of its molecular biology since its genome was first sequenced more than 10 years ago. In fact, a large amount of genomic data for *S*. *cerevisiae* strains is available (Wei et al., 2007; Novo et al., 2009; Borneman et al., 2011, 2013): there are 28 assembled genome sequences (mainly in draft format), and 19 are available as unassembled sequencing reads. Moreover, 35 sequences are available through project-specific websites (Borneman et al., 2013). In **Table 2**, wine *S*. *cerevisiae *sequenced strains are reported.

**Table 2:** Some traits of *Saccharomyces cerevisiae* strains from different origins.

	Specific traits	Reference
**Geographical origin**
West African	Poor utilization of galactose	Warringer et al. (2011)
	Hypersensitivity to high temperatures	
European	High respiratory capability (ethanol growth)	
	Good proliferation in synthetic wine must	
	Tolerance to copper, tartaric acid, Na^+^ and Li^+^ cations	
Malaysian	Utilization of melibiose and mannitol	
North	Unable to metabolize maltose	
American	Tolerance to oxalic acid	
**Ecological niche**
Laboratory strains	High production of ethyl butyrate and acetate	Camarasa et al. (2011)
	Low amounts of isoamyl acetate and biomass	
Commercial strains	High biomass and low acetate production	
	Short fermentation times	
Bakery yeasts	Low production of acetate, succinate, and glycerol	
Sake	Good utilization of glycerol Proliferation in absence of biotin	Warringer et al. (2011)

Recently, Borneman et al. (2012) described the genome sequence of the thiol-releasing commercial wine yeast hybrid VIN7. They showed that VIN7 is an almost complete allotriploid interspecific hybrid of *S. cerevisiae* and *S. kudriavzevii* that contains a heterozygous diploid *S. cerevisiae* genome and a haploid *S. kudriavzevii* genome. Both parental strains showed a European origin; in particular, the *S. cerevisiae* portion of the VIN7 genome was closely related to wine yeast but distant from the commercial wine yeasts QA23 and EC118 (Borneman et al., 2012). The genomes of *S*. *cerevisiae*/*S*. *kudriavzevii* hybrid strains display a mosaic structure that likely resulted from selective pressures experienced over time (Querol and Bond, 2009).

A comparative genome analysis between *S. cerevisiae* industrial and laboratory strains highlighted how the environment influences genomic structure and helped to identify genomic loci involved in the regulation of industrial phenotypes. In particular, substantial conservation throughout a core set of genes was observed, whereas many other regions displayed nucleotide substitutions likely involved in diversification and specialization events (Borneman et al., 2008).

Gene transfer is an important aspect of yeast diversification and may play a major role in adaptation to the wine fermentation ecosystem. Novo et al. (2009) sequenced the complete genome of the diploid commercial wine yeast EC118. They identified 34 ORFs encoding proteins potentially involved in carbon and nitrogen metabolism, cellular transport, and the stress response that were absent from S288c. BLASTP analysis suggested that these genes specific for EC1118 were acquired from non-*S. cerevisiae* donors. In fact, the closest relatives to EC1118 were found to be in species belonging to two clades. The first contained the *Lachancea*, *Zygosaccharomyces*, *Kluyveromyces*, *Saccharomyces*, and *Eremothecium* genera, and the second species belonged to a large, recently reassessed clade containing *Debaryomyces*, some *Pichia*, and a number of medically important *Candida* species.

The yeast genome is quite small at only 12 Mb but is highly packed, with approximately 6000 genes distributed over 16 chromosomes. Additionally, it contains two small, cytoplasmatic genomes: mitochondrial DNA (mtDNA) and killer double-stranded RNA (dsRNA). The biological and genetic characteristics of *S*. *cerevisiae* have been recently reviewed by Landry et al. (2006). Briefly, *S*. *cerevisiae* is a diploid yeast with highly clonal reproduction. *S. cerevisiae* is also homothallic, which confers the ability of regenerating a diploid cell from a haploid and could be interpreted as a way of genome renewal. This mechanism may be responsible for the high rate (28%) of homozygote strains found in vineyards (Mortimer et al., 1994). Many studies have also described the aneuploidy of wine (Bakalinsky and Snow, 1990; Guijo et al., 1997; Nadal et al., 1999), beer or bread strains (Codon et al., 1998).

The story of *S. cerevisiae* populations on earth is lost in the mists of history, and despite over 70 years of research, the biogeography of *S*. *cerevisiae* remains elusive; in fact, little is known about its ecology, origin, evolution, and distribution in nature (Wang et al., 2012). Naumov et al. (2006) hypothesized that the most ancient population of *S*. *cerevisiae *originated from Malaysia. Ancient *S*. *cerevisiae* DNA was discovered in Chinese pottery jars (7.000–5.500 B.C.; McGovern et al., 2004; Stefanini et al., 2012). Moreover, ribosomal DNA from *S*. *cerevisiae* was also found in some wine jars in the King Scorpion tomb in Abydos, Egypt, indicating that this yeast was responsible for wine fermentation by at least 3150 B.C. (Cavalieri et al., 2003).

During the last 30 years, a large number of observations have demonstrated that the wine strains of *S. cerevisiae* are highly diverse. Thus, the occurrence of specific natural strains likely depends on numerous factors such as climate conditions, the geographical location of the vineyard, the ripeness of the grapes, the age of the vineyard, the soil type, the grape variety, the application of antifungals, and the technique used to harvest (Combina et al., 2005; Valero et al., 2005, 2007; Raspor et al., 2006; Nisiotou and Nychas, 2007; Chavan et al., 2009; Li et al., 2010; Cordero-Bueso et al., 2011). In fact, another study found a relationship between specific natural strains and a particular *terroir *(Frezier and Dubourdieu, 1992; Sabate et al., 1998; Lopes et al., 2002; Schuller et al., 2005; Valero et al., 2007). Thus, the following definition of vitivinicultural “terroir” was provided: “*Vitivinicultural “terroir” is a concept which refers to an area in which collective knowledge of the interactions between the identifiable physical and biological environment and applied vitivinicultural practices develops, providing distinctive characteristics for the products originating from this area*” (Resolution OIV/Viti 333/2010).

However, insufficient quantitative data are available to establish general conclusions on the influence of these factors on the evolution of the fermentative biota of a given viticultural region, and extensive biogeographical surveys over many years are necessary (Schuller and Casal, 2007). Discrimination at the strain level thus becomes a strategic activity for the wine industry because it may link territory, environment, and final products for wine valorisation.

The species present on intact, undamaged berries have been reported to mainly belong to the group of oxidative basidiomycetous yeasts such as *Hanseniaspora uvarum*, *Cryptococcus* spp., *Rhodotorula* spp., *Sporobolomyces* spp., and *Filobasidium* spp. as well as to the dimorphic ascomycetous black yeast *Aureobasidium pullulans* (Prakitchaiwattana et al., 2004; Barata et al., 2008, Barata et al., 2012). In contrast, the most relevant fermentative wine yeast, *S. cerevisiae*, only occurs at concentrations less than 10–100 cfu/g of berry (Fleet, 2003). This yeast is present in nature at very low concentrations. On the surface of undamaged berries, its concentration is lower than 0.1%, although it is easily found on berries damaged by birds or insects (24%), which represent approximately 1 in 1000 grapes. In any case, some authors (Mortimer and Polsinelli, 1999) have shown that a population of yeast that is the primary source of natural yeast in wine production exists on grapes. Moreover, data indicate that yeast populations on wine grapes increase from 10^2^–10^3^ cfu/g on immature berries to 10^3^–10^6^ cfu/g on mature berries. Insects and birds are important agents for the dispersal of yeasts in different habitats. Regarding the role of insects as a vector for *S. cerevisiae* cells, Mortimer and Polsinelli (1999) demonstrated the presence of a flow of *S. cerevisiae* cells between the natural environment and cellars; because this yeast is not an airborne, it needs a vector to move. In particular, Francesca et al. (2012) also highlighted that migratory birds may act as vector for *S. cerevisiae *cells, but they are not a “reservoir” because the yeast cells survive in the gut for only 12 h. Stefanini et al. (2012) isolated yeast strains from wasps, grapes, and fermentations from the same vineyard over a span of different months and years. The results obtained showed that these strains were more similar to each other than strains derived from other environmental and geographical locations. Wasps therefore may play a role in maintaining ecological diversity.

However, whether these strains participate in alcohol fermentation in the cellar is still controversial: some authors (Ciani et al., 2004) observed that only cellar strains were responsible for alcohol fermentation in vats, whereas others showed that ‘grapevine strains’ may be partially responsible for alcohol fermentation (Constanti et al., 1997; Gutiérrez et al., 1999; Le Jeune et al., 2006).

## MOLECULAR METHODS

By using the techniques developed by molecular geneticists, new phylogenetic relationships were recognized, the number of separate species groups was reduced, and the diversity within the groups was increased (McCullough et al., 1998). Moreover, molecular methods revived the study of biogeography and positively impacted the final interpretation of biogeographic patterns (Ramette and Tiedje, 2007). In particular, a better knowledge of the microbial ecology of local ecosystems is essential to understand the winemaking process and to generate products with a local character, thereby allowing the development of modern winemaking practices and the diversification of wine products. Grapevine cultivation and wine production spread throughout the Mediterranean Sea toward Greece (5000 B.C.), Italy (900 B.C.), France (600 B.C.), northern Europe (100 AD) and much later, to the Americas (1500 AD). There are approximately 7.5 million hectares of vineyards across the world, mainly concentrated within the earth’s temperate zones, and two million are located in Europe (OIV, Statistical Report on World Vitiviniculture, 2012).

In particular, many molecular methods allow the identification of *S. cerevisiae *at the strain level, and they are required not only to investigate the diversity of this species but also to select strains for use as pure cultures, a widespread practice in winemaking industries where strains contribute to a specific characteristic of the final product (Dequin, 2001; Suzzi et al., 2012).

Sipiczki (2011) highlighted that *S*. *cerevisiae* wine strains are polyclonal and that the clones can differ significantly in oenological performance and genotype. The genomes of yeasts are subjected to duplications, deletions, and rearrangements that may cause the acquisition of new functions and gene specialization (Cubillos et al., 2011). Some authors (Guijo et al., 1997; Nadal et al., 1999) highlighted that aneuploidy may be a method of yeast adaptation through the modification of the expression of some genes involved in this process (Legras et al., 2007). In any case, aneuploids (Infante et al., 2003; Bradbury et al., 2005; Legras et al., 2007; Lopandic et al., 2007), triploids (Cummings and Fogel, 1978; Takahashi, 1978; Thornton, 1986), polyploids (e.g., Takahashi, 1978; Bakalinsky and Snow, 1990; Guijo et al., 1997; Naumov et al., 2000, 2002) and rarely haploids (Lopandic et al., 2007) may be present in the natural yeast biota of fermenting wine (Sipiczki, 2011).

Some studies have shown that genomic variability depends on telomeric recombination, which is important for adaptation to new environments and different metabolic sources and to overcome environmental stress, and on the insertion of transposable elements. Transposable elements comprise ~3% of the total sequenced genome of *S*. *cerevisiae* S288c (Carreto et al., 2008).

Carreto et al. (2008) showed that wine strains differed dramatically from the reference laboratory strain in Ty element composition, whereas clinical strains were similar to S288C in Ty element composition. Thus, it is likely that clinical strains and S288C had a common ancestor, and the differences found in wine strains may be due to the selective pressures that affect particular regions of the genome in response to adaptation to the environment. In particular, the variable genes were involved in metabolic functions related to cellular homeostasis or transport of different solutes such as ions, sugars, and metals. To better understand the population structure of wine *S*. *cerevisiae* strains, ecological studies using a polyphasic approach in order to define the biogeographical patterns have been carried out: a strict collaboration between phylogeneticists and ecologists and the development of new statistical tools provide a more comprehensive understanding of the factors controlling the *S*. *cerevisiae* biodiversity and biogeochemistry. The main molecular methods used for biogeographical studies are reported in **Table 3**.

**Table 3:** Molecular approaches used for *S*. *cerevisiae *biogeographical studies.

Molecular methods	Origin	Reference
aCGH	Brazil, Italy, USA	Weiss et al. (1999); Edwards-Ingram et al. (2004); Dunn et al. (2005),^2012^; and Gerstein et al. (2006)
Genome sequence and functional annotation	USA, Japan, France, Italy, Germany	Kvitek et al. (2008); Cavalieri (2009); Muller and McCusker (2009a),^2011^; Rolland et al. (2009); Bullard et al. (2010); Lelandais et al. (2011); and Scannell et al. (2011)
PFGE	Spain, Japan, UK, USA, France, South Africa, Ivory coast, Italy, Switzerland, West Africa, Russia, Portugal, Germany, China	Schwartz and Cantor (1984); Johnston and Mortimer (1986);Vezinhet et al. (1990),^1992^; Bidenne et al. (1992); Frezier and Dubourdieu (1992); Briones et al. (1996); Egli et al. (1998); Goto-Yamamoto et al. (1998); Mesa et al. (1999); Povhe et al. (2001); Sipiczki et al. (2001, 2004); Sipiczki (2011); Perez-Ortin et al. (2002); Carro et al. (2003); Schuller et al. (2004); Antunovics et al. (2005); Dunn et al. (2005); Aa et al. (2006); and Wang et al. (2012)
*mt*DNA-RFLP	France, Italy, Portugal	Vezinhet et al. (1990),^1992^; Querol et al. (1994); Versavaud et al. (1995); Schuller et al. (2005); and Di Maio et al. (2012)
RAPD-PCR	Spain, Chile, Peru, Uruguay, France, Italy	Quesada and Cenis (1995); Cavalieri et al. (1998); Martinez et al. (2007); and Tofalo et al. (2007)
Microsatellites analyses	New Zeland, Vietnam, France, Belgium, Russia, Czech Republic, Spain, The Netherlands, China, Taiwan, Japan, Croatia, Australia, Portugal, Austria, Germany, Brazil, Spain, Ghana, Nigeria, Lebanon	Ness et al. (1993); Versavaud et al. (1995); Gallego et al. (1998); Hennequin et al. (2001); Bradbury et al. (2005); Legras et al. (2005); Schuller et al. (2005),^2007^; Ayoub et al. (2006); Schuller and Casal (2007); Liti et al. (2009); Muller and McCusker (2009b); and Richards et al. (2009)
δ sequences	Lebanon, China, Vietnam, Japan, Taiwan, USA, The Netherlands, Italy, France, Portugal	Ness et al. (1993); Legras and Karst (2003); Schuller et al. (2004),^2012^; Legras et al. (2005); and Franco-Duarte et al. (2011)
MLST	Lebanon, China, Vietnam, Japan, Taiwan, USA, Italy, France, Germany, Indonesia, Chile, Uruguay, South Africa, New Zeland	Ben-Ari et al. (2005); Fay and Benavides (2005); Aa et al. (2006); Ayoub et al. (2006); and Vigentini et al. (2009)

### CGH ARRAY-BASED COMPARATIVE GENOMIC HYBRIDIZATION

Comparative genomic hybridization (CGH) is capable of detecting loss, gain and amplification of copy number at the chromosome level. Detection of amplifications is known to be sensitive down to less than 1 Mb. Therefore, one must take into consideration that although CGH is sensitive to specific types of copy number gains, its resolution for regional deletions is more limited. The use of array CGH overcomes this limitation, with improvements in resolution and dynamic range, in addition to the ability to directly map aberrations to the genome sequence and improved throughput (Weiss et al., 1999). This approach has been recently applied to investigate the evolutionary importance of genome size in *S*. *cerevisiae* (Edwards-Ingram et al., 2004; Dunn et al., 2005, 2012; Gerstein et al., 2006). Dunn et al. (2012) used this technique to study copy number variations (CNVs) across subtelomeric regions, non-S288c genomic regions, retrotransposons, and the non-nuclear mtDNA and 2-mm plasmids of 83 *S*. *cerevisiae* strains isolated from different industrial and natural environments. The obtained clusters for the different types of features showed that most of the CNVs occurred either in subtelomeric regions or among the classes of transposable elements and that there were no commercial wine strains that appeared to be absolutely identical to each other. Thus, these CNVs did not produce any clear phylogeny, so it is likely that an active interchange of these regions occurred rather than separate lineages descending from isolated ancestors, suggesting that most of these strains are the result of interbreeding between industrial and wild strains.

### GENOME SEQUENCE AND FUNCTIONAL ANNOTATION

The genetic diversity of *Saccharomyces* strains can also be assessed using genome sequencing and functional genomic analysis of transcript profiles. These approaches are useful to aid in the understanding of speciation, life history variation, conditional fitness trade-offs and the long term maintenance of complex genomic variation (Scannell et al., 2011). Genome sequencing provides the most complete understanding of the genomic structure of an organism and allows wide comparisons to be made between related species. Scannell et al. (2011) improved the genome sequences of three species belonging to the *Saccharomyces sensu stricto* complex (*S*. *bayanus* var. *uvarum*, *S*. *kudriavzevii*, and *S*. *mikatae*) and compared them with the genomes of *S*. *cerevisiae* and *S*. *paradoxus*. They identified 5261 annotated protein coding orthologs across all of the studied species. Moreover, they found genes that had been lost in one or more lineages. Generally, the lost genes were derived from yeast genome duplications, suggesting that this phenomenon still influences yeasts and contributes to phenotypic differentiation. These authors also detected lineage-specific gains and found, in particular, two horizontal gene transfers from bacteria. These genes differentiated the analyzed species, indicating their involvement in speciation and adaptation. Other authors such as Rolland et al. (2009) have also identified the presence of horizontal gene transfers from bacteria, confirming that this phenomenon plays important functional and evolutionary roles.

To characterize the genomes of large numbers of individuals, microarray-based methods provide an alternative to DNA sequencing. This method allows the identification of conserved and non-conserved regions across microbial populations. The use of tiling arrays followed by analysis of the DNA region via polymerase chain reaction (PCR) is useful to determine whether the absence of hybridization is due to deletion of a chromosomal region or due to areas of large sequence polymorphism (LSP; Muller and McCusker, 2011). Muller and McCusker (2011) characterized the genome-wide distribution of LSPs in 88 *S*. *cerevisiae *strains of diverse geographical origins and source substrates using high-density tiling arrays. They showed that LSPs occurred in the subtelomeric regions of chromosomes, where they did not disrupt essential gene expression. Moreover, this study revealed the presence of introgressions. In particular, clinical strains contained *S*. *paradoxus* DNA fragments. In another study, Muller and McCusker (2009a) developed a multi-species-based taxonomic microarray consisting of features targeted to multiple orthologous genes from *S. cerevisiae, S. paradoxus, S. mikatae, S. bayanus, S. kudriavzevii, N. castellii, L. kluyveri*, and the closely related *Candida glabrata*. In particular, they studied 183 supposed *S. cerevisiae *isolates of diverse ecological and geographical origins. They again confirmed the existence of introgressions in wine strains, identifying four hybrids, one between *S. cerevisiae *and *S. bayanus *and three between *S. cerevisiae *and *S. kudriavzevii*. In addition, this approach allowed the detection of multiple introgressed *S. paradoxus *DNA fragments in the genomes of three different *S. cerevisiae* isolates.

Other researchers studied comparative transcript profiling to define the relationships among strains (Kvitek et al., 2008; Cavalieri, 2009; Bullard et al., 2010). Conservation of a transcriptional response indicates functional relatedness of the organisms under investigation (Lelandais et al., 2011). In fact, genome rearrangements can modify gene expression and alter phenotypes. Kvitek et al. (2008) measured whole-genome expression in 52 strains collected from different niches (industrial processes and human illnesses) in the presence of different stress conditions. Wine strains were able to grow in the majority of the tested conditions; for example, copper resistance was predominant in wine strains, suggesting that the use of copper in the vineyard strongly selected against strains that were copper sensitive (Kvitek et al., 2008). This evidence confirmed that the process of fermentation imposes a strong selective pressure and therefore is a powerful evolutionary force in the generation of diversity (Kvitek et al., 2008; Cubillos et al., 2011; Sipiczki, 2011).

### PULSED-FIELD GEL ELECTROPHORESIS

Wine strains generally have a large diversity in the number and size of chromosomes that can be observed by pulsed-field gel electrophoresis (PFGE) analysis, which separates chromosome-sized DNA molecules. This method was first described by Schwartz and Cantor (1984) and is still one of the most powerful tools to investigate the biogeography and speciation of this yeast in nature. Analysis of the chromosomes of wine yeast strains by PFGE demonstrated the presence of chromosome-length polymorphisms, which are derived from chromosomal rearrangements such as translocations and deletions (Carro et al., 2003). Carro et al. (2003) suggested that the subtelomeric plasticity of chromosome I, which contains several membrane-associated genes, may induce rapid adaptive changes of the yeast strains in response to specific environmental cues (substrates). The reciprocal translocation between chromosomes VIII and XVI generated the SSU1-R allele, which confers sulfite resistance to yeast cells and was described as the first case of adaptive evolution, likely occurring as a consequence of the use of sulfites as a preservative in wine production (Goto-Yamamoto et al., 1998; Perez-Ortin et al., 2002). Many authors have showed that karyotyping is more discriminative than other approaches for yeast typing because it is able to highlight polymorphisms in electrophoretic chromosomal profiles in natural *S*. *cerevisiae* populations from almost all wine-growing regions of the world (Johnston and Mortimer, 1986; Vezinhet et al., 1990, 1992; Bidenne et al., 1992; Frezier and Dubourdieu, 1992; Briones et al., 1996; Egli et al., 1998; Povhe et al., 2001; Schuller et al., 2004; Sipiczki et al., 2004; Antunovics et al., 2005; Aa et al., 2006; Wang et al., 2012).

This approach showed that strains isolated from the same fermentation generally differ in chromosomal length (Egli et al., 1998; Mesa et al., 1999; Sipiczki et al., 2001, 2004; Antunovics et al., 2005), indicating that clones with different sets of chromosomes propagate at the same time and in succession during fermentation (Sipiczki, 2011). Wang et al. (2012) applied this technique to type *S*. *cerevisiae* strains with different ecological and geographical origins to better understand the ecology of *S. cerevisiae*. The obtained results showed that a wide divergence of populations of wild *S. cerevisiae* exist and that this divergence is only marginally affected by human activity. Dunn et al. (2005) revealed the existence of a set of deleted or amplified genes common to wine and other industrial yeasts, and certain genes have been identified as a possible wine yeast signature, particularly genes encoding membrane transporters.

### mtDNA-RFLP

*Saccharomyces cerevisiae* mtDNA is characterized by an elevated mutation rate. In particular, base-substitution mutations and length polymorphisms can be highlighted by restriction fingerprinting of mtDNA using endonucleases with different target sites (e.g., *Dde*I, *Hinf*I, *Alu*I, and *Rsa*I). The reliability and discrimination power of this fingerprinting technique are similar to those of PFGE.

The use of mtDNA-restriction fragment length polymorphism (RFLP) revealed a wide range of polymorphisms in mitochondrial genomes and mitochondrial genes (Vezinhet et al., 1990; Querol et al., 1994; Versavaud et al., 1995; Lopez et al., 2003). This technique was used together with PFGE by Vezinhet et al. (1992) to study the evolution of *S*. *cerevisiae* strains isolated from different wine regions over 6 years. The study demonstrated that some strains were widely distributed in the studied areas and present over several years, indicating that they are endemic to that region. More recently, Di Maio et al. (2012) used this method to investigate the biodiversity of wine yeast populations isolated over several years from Sicilian wineries where commercial yeast strains have never been used. mtDNA-RFLP allowed the differentiation of 209 of 918 yeast strains. Schuller et al. (2005) performed a large-scale biogeographical survey on the genetic diversity of *S. cerevisiae* strains isolated from spontaneous fermentations and identified 297 different genetic patterns among 1620 strains isolated from 54 small-scale fermentations of grapes from three vineyards located in the Vinho Verde region (Portugal) during a 3 year period. Almost all of the obtained patterns were unique, showing the large biodiversity of *S*. *cerevisiae* in that region.**

### RAPD-PCR

This technique is based on the use of a single short primer (8–12 nucleotides) that amplifies “anonymous” DNA sequences and represents a powerful typing method for many yeast and bacterial species (Quesada and Cenis, 1995; Martinez et al., 2007; Tofalo et al., 2007). In fact, the annealing of the primer at several points allows the user to obtain a complex banding pattern that is specific for each strain (Ivey and Phister, 2011). This method was used by Cavalieri et al. (1998) to differentiate 166 *S*. *cerevisiae *strains isolated from Tuscany and Sicily, two Italian regions. In this case, random amplified polymorphic DNA (RAPD)-PCR allowed the recognition of 16 patterns, and only 10 were strain specific. Tofalo et al. (2007) used this approach to recognize genetically different *S. cerevisiae *strains, which were clustered in subgroups related to the four different wine-producing areas of the Apulia region (Italy). The obtained results showed that the genetic differences reflect the phenotypic biodiversity.

### MICROSATELLITE ANALYSES

Microsatellites, also known as simple sequence repeats (SSRs) or short tandem repeats (STRs), are repeating sequences of 1–6 base pairs of DNA that are characterized by a high level of polymorphism. They occur within many open reading frames but are even more frequent in non-coding regulatory regions. In *S*. *cerevisiae*, microsatellites have been described as abundant and highly polymorphic in length (Richards et al., 2009), and for this reason, they are used as a reproducible and portable typing method (Gallego et al., 1998; Hennequin et al., 2001; Schuller et al., 2004; Bradbury et al., 2005; Legras et al., 2005). Recently, an increasing number of microsatellites have been described for *S*. *cerevisiae*, with the aim of identifying the most polymorphic loci with a high allelic diversity that can be used for both strain identification and the establishment of strain geographical or technological origin. Several studies used this approach to type *S. cerevisiae* strains of different geographical origins (Ness et al., 1993; Versavaud et al., 1995; Gallego et al., 1998; Hennequin et al., 2001; Bradbury et al., 2005; Legras et al., 2005; Schuller et al., 2005, 2007; Muller and McCusker, 2009b). For example, Schuller and Casal (2007) analyzed six polymorphic microsatellite loci in 361 strains isolated from the Vinho Verde region in Portugal during the 2001–2003 harvest seasons. Fifty-two new alleles were identified in addition to the 41 alleles previously described (ScAAT1–ScAAT6). Recently, a database of 246 genotypes has been compiled that includes 78 commercial strains of wine yeast, a range of yeast isolates from New Zealand wineries, and natural yeast strains from around the world, including 35 that were recently sequenced (Liti et al., 2009). Regardless of the technique chosen, a combination of different techniques is necessary to obtain unambiguous results. For example, Schuller et al. (2004) showed that genotypes with the same microsatellite pattern (using six loci) can have different karyotypes. This group also identified a large number of variants of a commercial wine strain that had escaped to adjacent vineyards (Schuller et al., 2007). Ayoub et al. (2006) also found that genotypes that could not be resolved by microsatellite profiles were sometimes discriminated by interdelta PCR or by sequence analysis. In *S. cerevisiae* 84 minisatellites have been reported, but recently four tandem repeated motif of 135 bp or larger called megasatellites have been described (Rolland et al., 2010). They are found in paralogous *FLO1*, *FLO5*, *FLO9*, and *NUM*1 genes. These motifs could be as targets to measure evolutionary relationships at intra- and intergenic levels (Rolland et al., 2010).

### δ SEQUENCES

The δ sequences are flanking sequences (300 bp) frequently associated with the Ty1 and Ty2 transposons that are dispersed throughout the genome and are particularly common in terminal chromosomal regions (Franco-Duarte et al., 2011). They are also found as single elements. The number (from 35 to 55) and the location of these elements are variable among species, so the δ sequences represent useful genetic markers for the identification of polymorphisms. Amplification of interdelta regions between neighboring d sequences generates strain-specific banding patterns. This method is suitable for the characterization of high numbers of strains because it is easy to perform, cheap and rapid. Recently, alternative primers (d12 and d21) that bind close to the initially described binding sites for primers d1 and d2 (Legras and Karst, 2003) were designed to improve this method (Ness et al., 1993). The combination of these primers (d12/δ21 or d12/d2) increased the discriminatory power of the method (Legras et al., 2005). In particular, the use of primer pairs d12/d2 showed the same discriminatory power as other methods, such as mtDNA, RFLP, microsatellite analysis, and karyotyping, for strain typing (Schuller et al., 2004). Schuller et al. (2012) used this approach to study the intraspecific genetic diversity of vineyard-associated *S*. *cerevisiae* strains. In particular, grapes were harvested from 16 vineyards over 2 years. A strict correlation between genotype and grape variety was found.

### MULTILOCUS SEQUENCE TYPING

Another technique used for *S*. *cerevisiae* strain typing is multilocus sequence typing (MLST), which was recently shown to be a powerful technique for typing microorganisms (Aa et al., 2006; Ayoub et al., 2006). Strains are characterized using the DNA sequences of internal fragments of multiple housekeeping genes where variation accumulates relatively slowly and tends to be selectively neutral. It is highly reliable and highly discriminatory at the strain level, and because it is based on nucleotide sequencing, the results are easily comparable between laboratories.

Recently, this technique was applied to study *S*. *cerevisiae* population structure and evolution (Fay and Benavides, 2005; Aa et al., 2006). Ayoub et al. (2006) tested a set of seven loci of 84 *S*. *cerevisiae* strains of different origins: 65 strains were isolated from traditional wineries in Lebanon, and the others were commercial wine strains and Asian isolates. MLST profiling allowed the differentiation of the Asian group of strains from the Lebanese and European commercial strains that appear closely related, suggesting the introduction of genetic material from Asian strains into Lebanon.

Vigentini et al. (2009) studied the genetic biodiversity of an *S. cerevisiae* collection including 33 commercial strains, 14 wine isolates, and three laboratory strains by screening for single-nucleotide polymorphisms (SNPs) in loci on genes involved in wine production. In particular, they focused on the identification of SNPs as new genetic markers. Several studies report the efficacy of this analysis for studying the evolution of a microbial population (Ben-Ari et al., 2005; Aa et al., 2006). The obtained results showed that the collection was characterized by a low polymorphism rate and degree of heterozygosity and that the gene coding for the trehalose-6-phosphate synthase enzyme, which is involved in ethanol resistance, could be used as a molecular target. In fact, this gene showed a sequence diversity of 1.42% with seven different nucleotide substitutions.

## CONCLUSION

Biogeographical studies revealed that *S*. *cerevisiae* species consists of both “domesticated” and “wild” populations which are phylogenetically distinct. These populations probably derives from the whole-genome duplication of a common ancestor strain. In particular, for *S*. *cerevisiae* wine yeast a clear geographical origin was established at regional and global scales suggesting that the different strains evolved independently for long time. They modified the dosage of some genes important for the persistence in specific ecological niches which represent a reservoir of natural yeasts. Comparative genomics studies highlighted that *S*. *cerevisiae* wine strains differ not only for their origin but also for genetic transfers from other yeasts (*Saccharomyces* and non-*Saccharomyces*) and bacteria. The main difference have been found in genes encoding cell wall proteins and associated with aminoacid uptake which are important for the production of sensorially volatile aroma compounds. So, the genomic tools are crucial to better understand genetic and molecular basis of yeast evolution and the “art” of wine making, so characterization of other yeast environmental isolates will be useful to develop tailor strains to meet consumer demand.

## Conflict of Interest Statement

The authors declare that the research was conducted in the absence of any commercial or financial relationships that could be construed as a potential conflict of interest.
